# Wings, Horns, and Butterfly Eyespots: How Do Complex Traits Evolve?

**DOI:** 10.1371/journal.pbio.1000037

**Published:** 2009-02-24

**Authors:** Antónia Monteiro, Ondrej Podlaha

## Abstract

Do novel complex traits evolve when pre-existent complex developmental networks are recruited into new places in the body? Here we look closely at the genomic fingerprints that are produced as a result of gene network co-option.

*“If we take modularity at all seriously, then any attempt to use developmental mechanisms as phylogenetic tools is doomed: how could one hope to distinguish between bona fide conservation (a stable history between mechanism character) and re-use or (worse yet) re-invention?”*—von Dassow and Munro, 1999 [1]

Throughout their evolutionary history, organisms have evolved numerous complex morphological, physiological, and behavioral adaptations to increase their chances of survival and reproduction. Insects have evolved wings and flight, which allowed them to better disperse [2], beetles have grown horns to fight over females [3], and moths and butterflies have decorated their wings with bright circles of colored scales to scare off predators [4]. The way that most of these and other adaptations first evolved, however, is still largely unknown. In the last two decades we have learned that novel traits appear to be built using old genes wired in novel ways [5], but it is still a mystery whether these novel traits evolve when genes are rewired de novo, one at a time, into new developmental networks, or whether clusters of pre-wired genes are co-opted into the development of the new trait. The speed of evolution of novel complex traits is likely to depend greatly on which of these two mechanisms underlies their origin. It is important, thus, to understand how novel complex traits evolve.

So far, our understanding of how adaptations and novel morphological traits are acquired is mostly founded on single gene case studies. On the one hand, researchers have focused on changes to morphology that involve regulatory or structural mutations in enzyme-coding loci predominantly at the termini of regulatory circuits (see reviews in [6,7]), and, on the other hand, researchers have dissected the genetic and/or developmental changes that underlie the modification or disappearance of pre-existent complex traits (Table 1). Few studies, however, have tried to directly address the genetic and developmental origins of new complex traits.

**Table 1:**
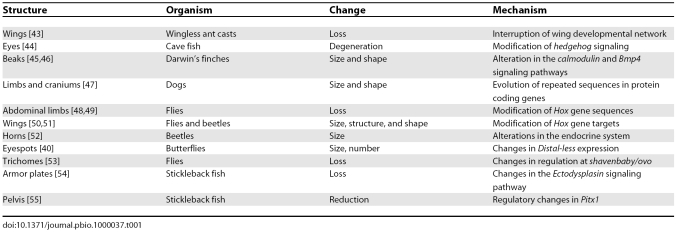
Examples of Complex Structures That Were Lost or Modified through the Course of Evolution

## How Do Functional New Complex Networks Evolve?

Complex traits require co-ordinated expression of many transcription factors and signaling pathways to guide their development. Creating a developmental program de novo would involve linking many genes one-by-one, requiring each mutation to drift into fixation, or to confer some selective advantage at every intermediate step in order to spread in the population. While this lengthy process is not completely unlikely, it could be circumvented with fewer steps by recruiting a top regulator of an already existing gene network, i.e., by means of gene network co-option. Subsequent modifications of the co-opted network could further optimize its role in the new developmental context.

**Figure 1:**
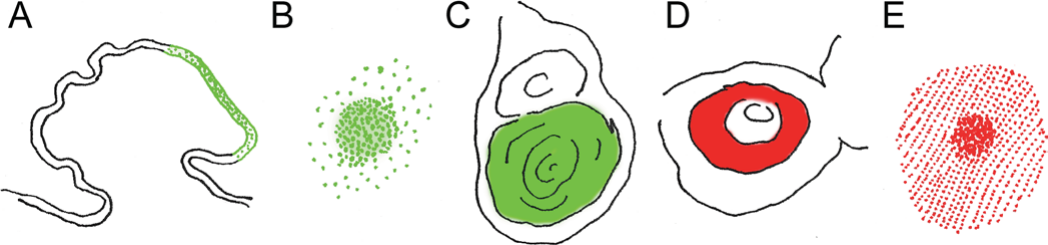
Similar Gene Expression in Various Non-Homologous Structures The transcription factor Distal-less (Dll) is expressed (A) in the horn primordium of the African dung beetle, Onthophagus nigriventris(modified from [9]); (B) in the eyespot focus of the squinting bush brown butterfly, Bicyclus anynana (modified from [40]); and (C) in the leg imaginal disc of the fruit fly, D. melanogaster (modified from [41]). The transcription factor Spalt (Sal) is expressed (D) in the antenna imaginal disc of D. melanogaster (modified from [42]); and (E) in the eyespot field of B. anynana pupal wings (modified from [12]).

Gene network co-option is hardly a new concept, but surprisingly, no test has ever been developed to distinguish it from alternative mechanisms. While gene network co-option is often proposed to explain instances where the same set of developmental genes are expressed in two different developmental contexts [8–14], de novo network evolution remains a feasible possibility that is rarely considered because of the perceived difficulty in distinguishing between these two alternative mechanisms (see quote above). For instance, the gene *Distal-less* (*Dll*) appears to specify insect distal limbs [15], the distal end of adult beetle horns [9], and the center of butterfly eyespots [10,16]. The ligands *decapentaplegic* and *wingless* are expressed both upstream as well as downstream of *Dll* during leg development [17], and orthologues are expressed also upstream and downstream of *Dll* expression in butterfly eyespot development [10,12]. The *spalt* gene is also present in both antennae and eyespots [18,19], downstream of *Dll* expression (Figure 1). Given that two non-homologous traits appear to share similar genes, and similar temporal expression patterns, there are two alternative ways of interpreting this data: (1) the same gene network was coopted into the production of the two non-homologous traits, or (2) for each of these developmental contexts a similar network was built independently using the same genes. Empirically distinguishing between these two alternatives is crucial if we are to propose that novel complex traits don't have to be put together gradually, gene by gene, but perhaps originate by the recruitment of pre-existent and functional developmental modular gene networks that operate in a different developmental context in the same organism.

## How Can We Attack the Problem?

Here we propose an empirical test that will help distinguish instances of gene network co-option from de novo network evolution. We propose that these different modes of evolution generate a different number of *cis*-regulatory elements (CREs) in genes belonging to the network. CREs link genes together into developmental networks and can provide information about the evolutionary history of the network in question. Below we detail a conceptual framework proposing that when gene network co-option occurs, the CREs that link genes downstream of top regulators, i.e., genes that sit at the top of a regulatory hierarchy, should continue to function in multiple developmental contexts, i.e., should be pleiotropic. This, however, is not the case when the network is built de novo. Although the implicit assumption in developmental genetics has been that one CRE drives gene expression in a particular pattern and in a particular developmental context, the concept of gene network co-option, when examined closely, challenges this assumption.

## How Can Modular Gene Networks Help Evolve New Complex Traits?

**Figure 2:**
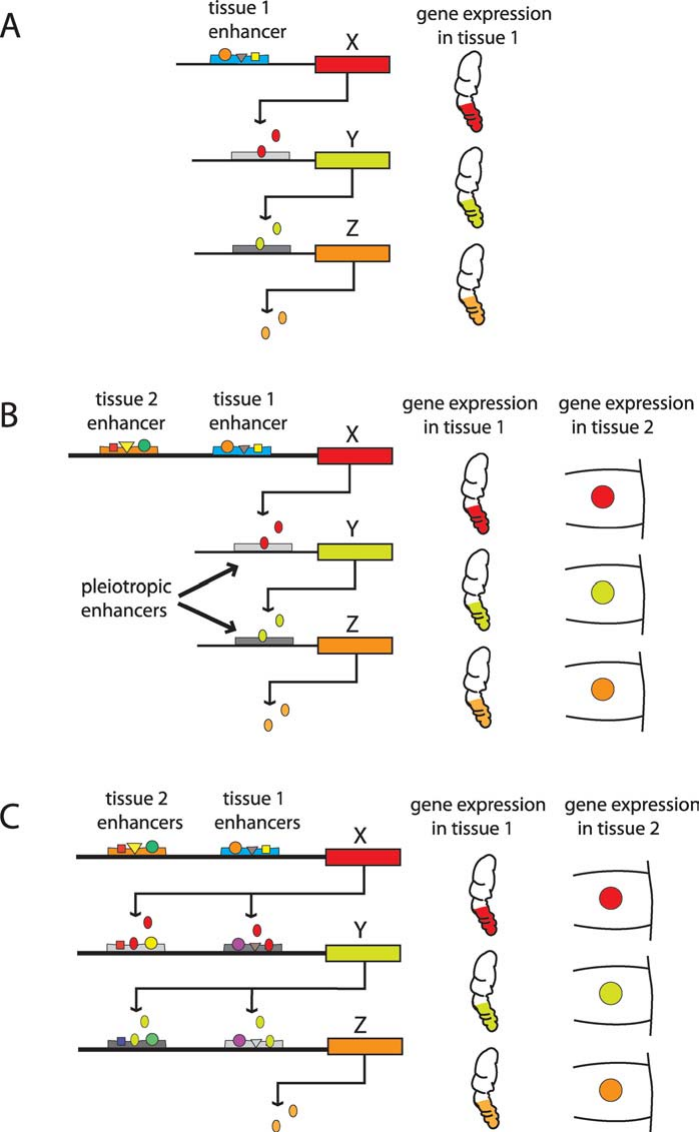
Hypothetical Illustration of Gene Network Co-Option and De Novo Network Evolution (A) Modular gene network where gene *X*, at the top of a regulatory network, directs expression of gene *Y*, which in turn directs expression of gene *Z*. All these genes are expressed in the tip of an appendage (e.g., leg) depicted on the right. (B) The modular gene network is co-opted into a new tissue by the evolution of a novel CRE in gene *X*. The *Y* and *Z* genes, which only receive inputs from *X* and *Y* genes, respectively, are also turned on in the new tissue (e.g., eyespot centers in a butterfly larval wing). The CREs of the *Y* and *Z* genes now have a dual function in directing gene expression in two separate developmental contexts, e.g., they are pleiotropic. (C) De novo network evolution where elements of the same non-modular gene network, *X*, *Y*, and *Z*, each evolve a separate CRE that drives gene expression in the novel developmental context.

We believe that for gene network co-option to occur the organism must have previously evolved modular gene networks. According to [5], a gene network consists of two or more linked gene regulatory circuits, which in turn consist of a signaling pathway (e.g., the *wingless* signaling pathway) that targets a particular gene. A modular gene network, on the other hand, is a network that behaves in an integrated and context-independent fashion during development [20]. Currently there is no knowledge regarding the size of these modular networks and how common they are in developmental networks, but we will exemplify the concept of modularity with a simple network consisting of two linked gene circuits (*X* activating *Y*, and *Y* activating *Z*, via their respective signal transduction pathways). The following simplified scheme omits the signal transduction elements: gene *X*, activated by a CRE in a particular developmental context, produces a protein that activates gene *Y* by binding to a CRE in *Y*, which in turn produces a protein that activates gene *Z* (Figure 2A). This simple network is a modular gene network because genes *Y* and *Z* are only receiving inputs from *X* and *Y*, respectively, but not from other regulators, and are thus able to work in a context-insensitive fashion. This means that if *X* is recruited into a novel developmental context (by the evolution of a novel CRE in *X*, for instance), then its connections to *Y* and *Z* are pre-made, and the latter genes may also be regulated in the new context.

Two examples of modular gene networks are beautifully illustrated in experiments with Drosophila. The gene *eyeless*, as well as three other top regulators, are able, when ectopically expressed in any imaginal disc (wings, antenna, legs), to differentiate clusters of eye units, or ommatidia, at those novel positions in the body [21,22]. This implies that genes downstream of these top regulators must be integrating or “reading” a different type of positional information (in their CREs) relative to their upstream activators. The CREs of genes in the middle of the eye network must be only receiving input or “listening” to their upstream regulators in the modular eye network, irrespective of the network's spatial deployment in the body. The same appears to be true for the leg network. Ectopic legs can develop by the ectopic expression of *Distal-less* [17] in leg imaginal tissue, flanking the normal expression domain for this gene. Again, if activating *Dll* is sufficient to initiate a leg program (within the molecular context of an imaginal disc), this implies that the set of transcription factors providing the positional information achieved up to the point of *Dll* expression are no longer necessary for the continuation of the rest of the leg developmental program. The genes downstream of *Dll* will be activated in cells in a context-independent way, irrespective of the cell's exact position within the imaginal disc. Again, this implies that the CREs of genes downstream in the leg developmental cascade are receiving a different type of input that does not necessarily contain precise positional information for the field of cells where legs develop. Instead, these CREs appear to integrate regulatory information from transcription factors belonging only to the modular leg network.

## How Do We Identify Gene Network Co-Option?

When observing the same set of orthologous genes being expressed in a similar temporal fashion in more than one developmental context and/or having the same function (being upstream activators of *X*, downstream repressors of *Y*, etc.), it is natural to question whether there was a network co-option event, or whether the genes were re-wired de novo to each other in the novel context. By examining the “wiring details” of the putative internal network genes, such as genes *Y* and *Z* in the hypothetical example above (Figure 2), we may be able to discriminate between the two scenarios. If the network was co-opted via the evolution of a new CRE in a top regulator (gene *X*), allowing genes *Y* and *Z* to also be expressed in the novel context, then the CREs of genes *Y* and *Z* should be pleiotropic and function in the two different developmental contexts (Figure 2B). Finding such a pleiotropic CRE would lend strong support for a simple genetic mechanism by which complex (but context-insensitive) gene regulatory networks can be co-opted by recruitment of a top regulator into a new spatial-temporal context. On the other hand, if modular networks are not common features of developmental systems, then activating the internal network genes, *Y* and *Z*, in the precise temporal-spatial positions depicted in Figure 2C requires the evolution of complex and dedicated CREs for each developmental context. These CREs would integrate the input of upstream regulators as well as additional positional information specific to that tissue or organ. Once a network is built in tissue 1, it cannot be automatically co-opted into tissue 2 because the CREs that drive gene expression in context 1 are unable to do so in context 2 (Figure 2C). Additional CREs have to evolve de novo, probably one at a time, in order to rebuild the same network in tissue 2. In this scenario we do not expect to find pleiotropic CREs.

## How Do We Test Whether a CRE Is Pleiotropic?

The current method used to test whether a stretch of DNA has regulatory function is to place it in front of a reporter gene, such as *EGFP* or *LacZ*, and observe the patterns of reporter gene expression that are formed during development. These experiments are usually time consuming because they involve attaching multiple putative regulatory DNA fragments to reporter constructs in vitro, and then generating transgenic lines to test reporter gene expression in vivo. Because the main goal of these experiments has been to identify CREs for single developmental contexts, there are currently little data available that can be used to directly test the ideas presented here (but see [23] for an example of network co-option across species). The examples we gathered below are usually mentioned as side observations to the main goals of the papers. By observing that multiple expression domains of a gene, driven by the same CRE, could represent instances of gene network co-option, we are hoping to bring attention to the additional uses of a transgenic line containing reporter constructs once a CRE is found. Conversely, a construct that drives a reporter in only one of the gene's multiple expression domains indicates that the deployments of the gene have likely evolved independently.

## What Data Support the Existence of CREs Acting in Multiple Developmental Contexts?

Several studies have experimentally identified CREs that appear to control gene expression in multiple developmental contexts; sometimes in serial homologous traits, but often in non-homologous traits as well. Gene expression in the latter traits, but not the former, supports a gene network co-option scenario.

**Figure 3:**
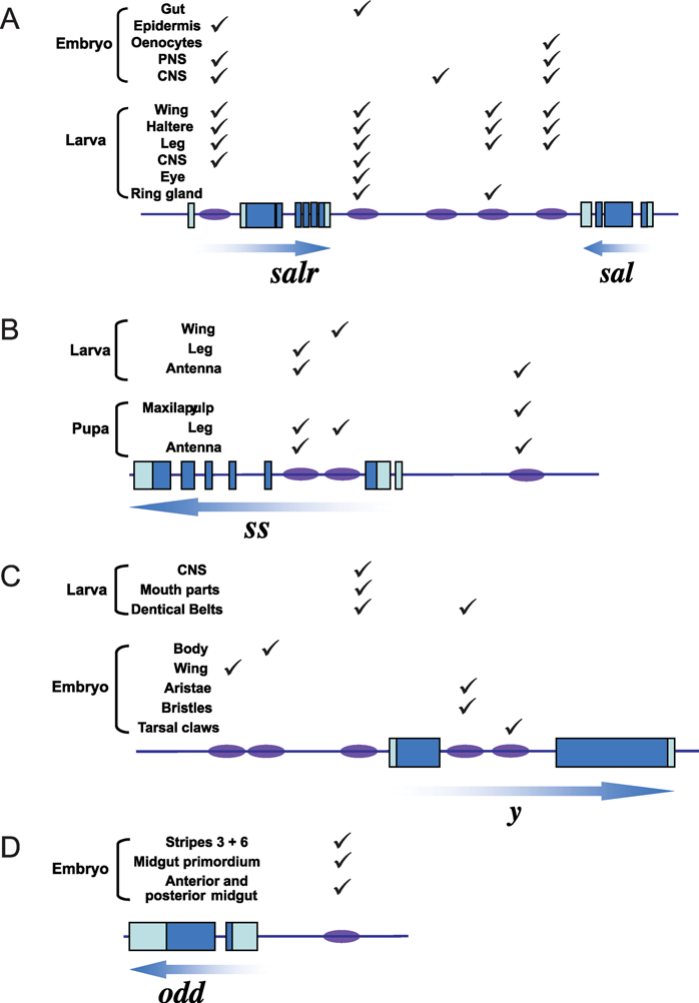
Examples of Pleiotropic CREs A schematic representation of putatively pleiotropic CREs is shown for: (A) The *spalt* (*sal* and *salr*) gene complex; (B) *spineless* (*ss*); (C) *yellow* (*y*); (D) *odd-skipped* (*odd*). Gene orientation is marked by arrows. Ovals show approximate position of CREs surrounding the protein-coding genes. Checkmarks of tissue/organs above CREs represent the multiple domains of gene expression driven by the same CRE. Modified from [24,26–28]. The multiple CREs that drive gene expression in the same tissue or organ mostly drive gene expression in distinct cell populations. Abbreviations: CNS, central nervous system; PNS, peripheral nervous system.

For instance, Barrio et al. [24] analyzed the region surrounding the *spalt* (*sal*) complex locus and found several CREs that drove reporter gene expression in multiple Drosophila tissues and organs and during different developmental stages (Figure 3A). For example, one fragment was found to drive expression in the embryonic gut, larval wing, haltere, leg, central nervous system, eye, and ring gland. It is interesting to note that a portion of the *sal* complex CREs drove reporter gene expression in the leg, where *sal* is usually not expressed. A simple explanation for this observation is that the CRE tested was fragmented in such a way that potential suppressor sites (binding sites to Antennapedia [25]) might have been excluded from the construct, and thus the CRE fragment was able to turn on a reporter gene where *sal* is normally not observed.

Conversely, it is also possible that when a regulatory piece of DNA is tested in vivo, one cannot be sure that it does not contain multiple independent CREs, adjacent to each other, each regulating the gene in a separate developmental context. Only additional experiments that dissect the enhancers further, to find minimal regulatory sequences for each developmental context, would suffice to unequivocally show overlapping CREs.

If overlapping CREs are found, however, it is still possible to imagine that the same piece of DNA could contain mutually exclusive sets of binding sites for transcription factors that drive gene expression in different tissues. This scenario, though possible, is unlikely because the de novo binding sites would have to evolve on top of an existing CRE (a few hundred nucleotides) out of all the regulatory space available (possibly tens of thousands of nucleotides). If overlapping CREs were indeed to occur (we currently don't know of any examples), then only a functional disruption of combinations of binding sites would show that this seemingly “pleiotropic” CRE in fact isn't, and does not represent an instance of gene network co-option.

Additional examples of CREs that drive gene expression in multiple contexts include (1) CREs in the gene *spineless* (*ss*) in Drosophila, which are able to drive reporter gene expression during embryonic and pupal stages and in different imaginal discs [26] (Figure 3B); (2) a CRE in the upstream regulatory sequence of *yellow* (*y*) of D. melanogaster that drives *yellow* expression both in the developing larval central nervous system and in the larval mouth parts and denticle belts [27], and is able to alter both male mating behavior as well as pigmentation in the latter larval structures when disrupted (Figure 3C); and (3) a CRE in *odd-skipped* (*odd*), a gene involved in Drosophila segmentation, which drives expression of a reporter gene first in two segmentation stripes early in embryogenesis and later in different populations of cells of the mid- and hindgut (Figure 3D) [28].

All of the data above suggest that a network co-option event took place by the deployment of an upstream regulator, of the featured internal network gene, into a novel developmental context, resulting in the creation of multifunctional (and pleiotropic) CREs.

## What Happens after a Network Is Co-Opted?

When a network co-option event takes place, it is reasonable to assume that some genes may not be activated in a similar way (perhaps co-factors are missing from the new developmental context), or that they may not be able to activate the exact same set of downstream targets. However, it is also possible that some downstream effector genes will still function in the novel context, and a phenotype will emerge. Given enough time, the network may be further modified by the addition of novel downstream targets, and elimination of old ones. Conversely, it is also plausible that genes co-opted as part of a larger network, and no longer functional in the new developmental context, maintain their CRE sequences intact. This would happen because purifying selection would be acting on the common CRE that is driving functional gene expression in the ancestral development context. For instance, in D. santomea there is a functional CRE in the *yellow* gene (it can drive reporter expression in the last two abdominal segments of D. melanogaster) despite the current absence of Yellow protein in those segments (due to evolution of trans factors in D. santomea) [29]. Is this element conserved in D. santomea because a network involving the internal network gene *yellow*, regulated by this CRE, was co-opted into a new developmental context, and this CRE is serving another developmental function? If further analyses of this CRE indicate a functional role in a different developmental context, it would support the hypothesis of gene network co-option.

It is also possible that after a network co-option event, molecular mechanisms such as CRE duplications followed by sub-functionalization take place. These mechanisms would allow natural selection to further optimize duplicated CREs to the trait's specific function and potentially obscure the evolutionary origin of the novel trait. Because of these mechanisms, it is likely that our experimental method will perform better when applied to genes involved in building complex structures of recent origin.

## What Data Support the Existence of De Novo Network Evolution?

In order for two similar gene networks to be considered non-homologous, it is important to establish that the networks are similar because they use orthologous rather than paralogous genes, and that the same functional relationships between the genes are in place, before examining the network wiring details using our proposed framework. The eye developmental network, for example, failed to meet the criteria above for some of its component genes, suggesting that these genes were wired de novo in separate lineages. Networks were compared across organisms, rather than within the same organism, as proposed in our framework, but the essential question remained the same—whether the two eye networks derived from a common ancestral network. Comparisons of the eye network in insects and vertebrates showed instead that despite similarities in the genes that are part of these networks, some genes are not orthologous but paralogous copies, and the functional relationships between them are different (reviewed in [30,31]). Because the paralogous genes (*sine oculis* in Drosophila, and *Six3* and *Optx2* in Xenopus) were present long before insects and vertebrates diverged, it is more parsimonious to assume that each was recruited separately into the eye network in each lineage, rather than that the two copies (and associated CREs) were maintained as duplicate and redundantly expressed eye genes for long enough to allow a different paralogue to be silenced in each lineage. As such, probing the CREs belonging to these genes in the network appears to be no longer necessary to show de novo re-wiring.

## What Are the Evolutionary Implications of Pleiotropic CREs?

Biologists disagree over what kind of genetic change is mostly responsible for adaptive evolutionary change [5,6,32,33]. There are two main proposed regions where change can take place: in regulatory DNA, and in protein-coding DNA. The argument centers on which of these regions suffers from the least amount of pleiotropy. Defenders of the non-coding DNA region argue that the evolution of modular CREs allows a gene to evolve new functions without impairing its old functions, because change in protein sequence is not needed [5,34]. Evidence is now mounting, however, that proteins themselves, including Hox proteins, are highly modular, i.e., different parts of the protein bind to different co-factors (required for joint gene regulation in only a sub-set of developmental contexts), and alterations in the protein sequence may affect one of its functions but not all of them simultaneously [33]. Here we have presented a conceptual framework that also weighs on this controversy. In particular, if gene network co-option is commonplace in the differentiation of new organs and tissues, an arguably large number of CREs, belonging to genes in the middle of modular networks, may be found to be highly pleiotropic and under strong stabilizing selection—behaving quite similarly, in fact, to protein coding sequences, at least in our earliest understanding of them. This new framework suggests that the discussion about protein or regulatory sequences playing the larger role in adaptive evolution is too simplistic and possibly misguided. Both protein-coding and regulatory sequences can be modular and pleiotropic. The discussion should focus instead on the evolution of pleiotropic versus “modularized” genomic regions traits, on whether or not modular networks are common in developmental systems, on the size of these networks, and on the role that gene network co-option has played in structuring the regulatory regions of genes.

Additionally, gene network co-option also weighs on the efforts to annotate non-coding genomic sequences [35], and attempts to characterize gene expression in response to environmental factors [36,37]. The conceptual framework of gene network co-option illustrates that for a battery of genes to be expressed in a particular co-ordinated fashion during development, or in response to an environmental stimulus, they do not all need to have temporal or positional information encoded in the same way in their CREs, i.e., they do not need identical motifs within CREs. This information, however, is often disregarded in the field of microarray genomics (but see [38]), or in efforts to annotate non-coding sequences. In recent papers, it is usually assumed that the regulatory sequences of all genes expressed in a particular tissue or in response to a certain environmental stimulus should contain an enrichment of binding sites for the same transcription factors [35–37]. This approach does not account for the concept of a modular regulatory hierarchy where a first tier of genes respond to the stimulus, and a second tier of genes respond only to the first tier of genes. The point is that CREs containing different types of transcription factor binding sites can regulate genes in the same spatial area, or in response to the same environmental stimulus.

## Toward a Solution

There is still much to do in order to fully understand how novel complex traits evolve. Here we propose an experimental framework that will allow us to test whether novel traits evolve from pre-existing genetic networks. In order to do this, it is important to continue exploring the full complement of genes that are shared across multiple traits to identify gene clusters that may be behaving as an integrated and context-insensitive network of genes [20,39]. For those traits that appear to share a common network, it is then important to ask whether the similarities extend to a shared regulatory hierarchy and function within the network. If this is the case, then the next step is to target genes that appear to be in the middle of the putatively shared network and look at their wiring details. The CREs of these genes, identifiable with transgenic tools, should be tested for multiple expression domains across a variety of developmental contexts where network co-option is suspected. If the same CRE drives gene expression in the different structures, network co-option is the likely mechanism; if they don't, the network was likely built de novo.

This work is difficult and time consuming, but the question at its core—the genetic origin of new and complex traits—is probably still one of the most pertinent and fundamental unanswered questions in evolution today. At stake is the possibility of testing whether novel complex traits arise from a gradual building of novel developmental networks, gene by gene, or whether pre-existent modules of interacting genes are recruited together to play novel roles in novel parts of the organism.

## Abbreviations

CRE: *cis*-regulatory element
